# Knockdown of SLC7A5 inhibits malignant progression and attenuates oxaliplatin resistance in gastric cancer by suppressing glycolysis

**DOI:** 10.1186/s10020-025-01175-9

**Published:** 2025-03-25

**Authors:** Yan Zhang, Jian Cao, Zheng Yuan, Jiahui Zhou, Hao Zuo, Xinsheng Miao, Xinhua Gu

**Affiliations:** 1 Department of Gastrointestinal Surgery, Suzhou Municipal Hospital, Suzhou Hospital Affiliated to Gusu School of Nanjing Medical University, Suzhou, 215000 China; 2 Department of Gastroenterology, Suzhou Municipal Hospital, Suzhou Hospital Affiliated to Gusu School of Nanjing Medical University, Daoqianjie 26, Suzhou, 215000 China

**Keywords:** Gastric cancer, Oxaliplatin resistance, SLC7A5, Glycolysis, Bioinformatics

## Abstract

**Background:**

Chemotherapy resistance is a major challenge in the treatment of intermediate and advanced gastric cancer (GC). This study aimed to recognize oxaliplatin resistance-related genes (OXARGs) in GC and to explore their role and mechanism in oxaliplatin resistance of GC.

**Methods:**

OXARGs with prognostic value in GC were analyzed using GC oxaliplatin resistance data from the GEO and TCGA databases. RT-qPCR and WB assay were applied to verify the expression of MT2A, NOTCH1 and SLC7A5 in oxaliplatin-resistant GC cells (HGC27R and MKN45R). The effect of SLC7A5 on the malignant phenotype of oxaliplatin-resistant GC cells was verified by CCK-8, EDU, TUNEL, colony formation, wound healing, transwell assay, tumor bearing experiments and WB assay.

**Results:**

Bioinformatics analysis and experimental validation indicate that SLC7A5 was a target for oxaliplatin-resistance in GC. Knockdown of SLC7A5 obviously decreased the viability, migration, and invasion of oxaliplatin-resistant GC cells in vitro and tumor growth in vivo. It also increased the apoptosis levels and BAX expression, and reduced the expression of BCL2, MMP 2 and MMP9. Additionally, the knockdown of SLC7A5 enhanced the sensitivity of oxaliplatin-resistant GC cells to oxaliplatin both in vitro and in vivo. Furthermore, knockdown of SLC7A5 downregulated the expression of HK2, LDHA, Glut1, and PDK1 both in vivo and in vitro, leading to increased extracellular glucose levels and decreased lactate levels. However, glutathione significantly attenuated the regulatory effect of SLC7A5 knockdown on the malignant phenotype of oxaliplatin-resistant GC cells.

**Trial registration:**

Not Applicable.

**Conclusion:**

Knockdown of SLC7A5 inhibits malignant progression and attenuates oxaliplatin resistance in GC by suppressing glycolysis.

**Supplementary Information:**

The online version contains supplementary material available at 10.1186/s10020-025-01175-9.

## Introduction

Gastric cancer (GC) is a common malignant tumor globally, ranking fourth and fifth in morbidity and mortality among all cancers, respectively (Sung et al. 2021). With advancements in early screening and treatment methods, the postoperative survival rate of early-stage GC patients has improved (Ryu et al. 2022). However, for advanced or recurrent GC, chemotherapy remains one of the main treatment strategies (Luo et al. 2021). Oxaliplatin is a widely used chemotherapy drug GC treatment, exerting significant therapeutic effects on various malignant tumors by inhibiting DNA synthesis (Zhang et al. 2022; Yang et al. 2021). However, with increasing use of oxaliplatin, more and more patients develop drug resistance, which severely affects the efficacy of oxaliplatin and patient prognosis (Petrioli et al. 2020). The emergence of resistance is usually caused by various mechanisms such as drug transport, drug metabolism, DNA repair, and changes in cell death pathways (Liu et al. 2024). Therefore, studying the mechanisms of resistance of GC cells to oxaliplatin is of great clinical significance and can bring out new ideas for the treatment of GC.

Bioinformatics, as an emerging interdisciplinary field, provides a crucial avenue for the search for cancer biomarkers. For instance, Yang et al. identified LINC01133 as a potential therapeutic target for metastasis in GC through bioinformatics analysis and experimental validation. The study found that LINC01133/miR-106a-3p inhibits GC epithelial-mesenchymal transition and metastasis by deactivating the Wnt/β-catenin pathway in an adenomatous polyposis coli (APC)-dependent manner (Yang et al. 2018). Another analysis revealed differential expression of POU5F1 in GC tissues, and researchers discovered that POU5F1 promotes the malignant progression of GC by decreasing the ubiquitination level of TRAF6 (Yang et al. 2023). Li et al. identified the prognostic value of ATXN2L in GC through bioinformatics analysis and experimentally confirmed that blocking EGFR/ATXN2L signaling can reverse oxaliplatin resistance in GC cells and inhibit migration (Lin et al. 2019). Similarly, based on bioinformatics analysis, this research identified that SLC7A5 was associated with oxaliplatin resistance in GC and with prognostic value. Previous research has shown that SLC7A5 acts as a downstream target of multiple signaling molecules and is involved in the proliferation and migration of GC cells (Wu et al. 2024; Ma et al. 2022; Wang et al. 2016). Nevertheless, the role of SLC7A5 in GC progression and whether it has played a role in oxaliplatin resistance in GC still needs to be studied.

Therefore, this study systematically combined bioinformatics technology and experimental methods to evaluate the role and potential mechanisms of SLC7A5 in oxaliplatin-resistance in GC both in vitro and in vivo. Through invasion, migration, apoptosis, and xenograft tumor model, the study confirmed the impact of SLC7A5 on the malignant progression of oxaliplatin-resistant GC cells. The aim is to elucidate the role of SLC7A5 in oxaliplatin resistance in GC, providing a reference for the diagnosis and treatment of oxaliplatin-resistant GC.

## Materials and methods

### Data collection and processing

GSE128967 (resistance = 7, control = 4) dataset related to oxaliplatin-resistance of GC was obtained from the GEO database. In addition, RNA sequencing results of 412 STAD patients with clinical information were obtained from the TCGA database and processed through the “TCGAbiolinks” R package. The platinum resistance-related genes (PRGs) were obtained from the Platinum Resistance database.

### Differentially expressed genes (DEGs) screening and enrichment analysis

Differential analysis in the GSE128967 dataset was performed using the R package “limma 3.58.1”. The threshold for differential gene screening were |log2 FC| > 1 and *P* < 0.05. The differentially expressed genes were chosen for GO and KEGG pathway enrichment using the “clusterProfiler” package, and the top 15 significant pathways with *P* < 0.05 were selected. Metabolic pathway enrichment was conducted based on PRGs using the GSEABase package, and the differential genes were analyzed through GSEA and adj. *P* < 0.05 were selected.

**Table 1:** The sequences of the primers

Gene	Primer	Sequences(5’-3’)
*β-actin*	Forward	GGAAATCGTGCGTGACATTAAG
	Reverse	AGCTCGTAGCTCTTCTCCA
*SLC7A5*	Forward	GTCCAATCTAGATCCCAACTTCTC
	Reverse	ATTCCATCCTCCATAGGCAAAG
*MT2A*	Forward	ATGCACCTCCTGCAAGAAA
	Reverse	AGCAGCTGCACTTGTCC
*NOTCH1*	Forward	GAGGACCTCATCAACTCACAC
	Reverse	CTCCCTGTTGTTCTGCATATCT

### Screening of oxaliplatin resistance genes with prognostic value

The oxaliplatin resistance-related genes (OXARGs) in GC were obtained by intersection of DEGs from GSE128967 dataset and PRGs. Based on the TCGA-STAD survival data, Kaplan-Meier (KM) curves were plotted using the survival package to identify OXARGs with prognostic value (*P* < 0.05). The risk score was determined using a linear combination of regression coefficients (α) and gene expression levels in a multivariate Cox regression model based on the “ggrisk” package. The prognostic value of the genes was assessed using KM survival curves and ROC curves. In addition, the drug sensitivity of each patient was predicted based on the RNA-seq results using the “oncoPredict” R package.

### Immune infiltration and CNV mutation analyses

The transposed convolution module of the “IOBR” package was used to estimate the tumor microenvironment (TME). TME integrates six transposed convolution methods, namely CIBERSORT, MCPcounter, EPIC, xCell, quantiseq, and TIMER. Selected significant immune infiltration prediction results (*P* < 0.01) to construct box plots and conduct immune infiltration correlation analysis on drug resistance-related genes. The “UCSCXenaTools” was employed to obtain TCGA-STAD mutation data, and analyze and visualize the mutation data using “IOBR”.

### Cell culture

MKN45 and HGC27 human GC cells (Cell Bank of the Chinese Academy of Sciences, Shanghai, China) were cultured in RPMI-1640 medium and added 10% FBS, penicillin, and streptomycin at 37 °C with 5% CO2. Oxaliplatin-resistant GC cells MKN45R were obtained from MEISEN CELL Life Technology Co., LTD (Zhejiang, China). Oxaliplatin-resistant GC cells HGC27R were generated by subjecting cells to increasing doses of oxaliplatin (MCE, USA) from 0.25 µg/mL to 2 µg/mL (Ren et al. 2023). Cells are passaged every 3 days and cultured for approximately 2 months at each concentration of oxaliplatin. The establishment of drug-resistant cell lines takes about 8 months. The drug-resistant strains can grow in oxaliplatin-containing medium at a rate no slower than that of wild-type cell lines in medium without oxaliplatin, which is considered a successful establishment of drug-resistant strains. Drug resistance was confirmed by cell counting kit-8 (CCK-8) assay.

### Cell transfection

The SLC7A5 sh-RNAs (sh-Y31243, 5′-GCATTATACAGCGGCCTCTTT-3′; sh-Y31244, 5′-CTAGATCCCAACTTCTCATTT-3′; sh-Y31245, 5′-GCCGTGGACTTCGGGAACTAT-3′) and its negative control (sh-NC, 5′-CCTAAGGTTAAGTCGCCCTCG-3′) were designed and obtained by OBiO Technology Corp.,Ltd. (Shanghai, China). The cells were transfected using Lipofectamine RNAiMAX Transfection Reagent (Invitrogen). Transfected cells were collected after 48 h and then assessed by RT-qPCR and western blotting (WB).

### CCK-8 assay

The GC cells were seeded in 96-well plates (3 × 10^3^ cells/well) and treated with different doses of oxaliplatin for 24 h. Afterward, 10 µL of CCK-8 reagent (Beyotime, China) was added into each well and incubated for 2 h. The absorbance was measured at 450 nm using a microplate reader.

### Reverse transcription-quantitative polymerase chain reaction (RT-qPCR) assay

Total RNA from GC cells was extracted using a vezol-pure total RNA isolation kit (Vazyme, China) and quantified using the NanoDrop spectrophotometer (Thermo Scientific). cDNA was reverse transcribed using the PrimeScript RT Master Mix (TaKaRa, Japan), and the cDNA samples were subjected to quantitative PCR using the Real-Time PCR system (Bio-rad, USA). RT-qPCR reaction conditions were as follows: 95 °C for 30 s, followed by 40 cycles alternating between 95 °C for 5 s and 60 °C for 30 s. Primers were listed below (Table 1). Relative mRNA expression levels were determined using the 2^−ΔΔCT^ method.

### 5-ethynyl-2- deoxyuridine (EDU) staining

GC cells were seeded into a 96-well plate (1 × 10^5^cells/well) and treated with 100 µL medium containing 50 µM EDU (E2051, Applygen, China) for 2 h. After glycine neutralization, cells were immobilized with 4% paraformaldehyde (BL539A, Biosharp, China) for 30 min and permeated in 0.5% TritonX-100 (E2051; Applygen, China) for 10 min. Cells were then stained with 4’, 6-diaminidine − 2 phenylindole (DAPI). The positive cells were observed by a fluorescent microscope (Nikon, Japan).

### Colony formation assay

The cells were counted after trypsinization and transfection for 48 h. 1 × 10^3^ cells were plated into a 6-well plate, and the culture medium was replaced every 3 days. After 14 days, the cells were stained with 0.05% crystal violet for 30 min. Then, the cell colonies were counted and analyzed.

### Wound healing assay and transwell assay

GC cells were plated into 6-well culture plates (1 × 10^5^ cells/well) and incubated until 80% confluence was reached. Then, the wounds in cell monolayers were made with a 200uL pipette tip, following which was the rinsed with PBS for the removal of cell debris. After 24 h incubation, photographs were taken to estimate wound healing. The pre-treatment of transwell chambers (Corning, New York, USA) with 0.1mL of matrigel (Becton Dickinson, USA) was implemented at 37 °C. Then, the suspension of collected cells at a final concentration of 2 × 105 cells/mL was conducted in serum-free DMEM. Following the injection of cell suspensions into the upper wells, the medium containing 5% FBS was placed in the lower chamber. After that, the invaded cells on the lower face were subjected to 100% methanol fixation as well as hematoxylin and eosin staining.

### TUNEL staining

A TUNEL Apoptosis Detection kit (Beyotime, China) was employed to assess the apoptosis of GC cells. GC cells were cultured on a 6-well plate (2 × 10^6^ cells/well) until the cells covered more than 70% and were fixed with 4% formaldehyde for 30 min. Then, the cells were washed with PBS and incubated with 0.5% Triton-X-100 for 20 min. Then, cells were incubated with 50 µL TUNEL buffer at 37 °C for 1 h in the dark. The apoptotic cells were detected by a fluorescence microscope (Olympus).

### Detection of glycolysis indicators

The glucose and lactic acid production was measured using the Glucose content detection kit (60408ES60, yeasen) and Lactic acid (LA) content detection kit (BC2235, solarbio). The the Seahorse XF Cell Mito Stress Test Kit and Glycolysis Stress Test Kit were employed to assess the oxygen consumption rate (OCR) and extracellular acidification rate (ECAR). 10 mM glucose, 1 µM oligomycin, and 100 mM 2-deoxyglucose were added automatically to assess the ECAR. 1 µM oligomycin, 1 µM carbonyl cyanide-4- (trifluoromethoxy) phenylhydrazone, and 1 µM rotenone were added automatically to XF-96 cell culture microplates to test OCR.

### Xenograft tumor model

SPF-grade BALB/C nude mice (18–22 g, 3 weeks old, female) were obtained from Hangzhou Ziyuan Experimental Animal Technology (Hangzhou, China). Each group of experiments used 6 mice for repetition. GC cells (1 × 10^6^) transfected with shRNA-NC or shRNA-SLC7A5 GC were injected subcutaneously into the mice. The mice were observed every two days for a period of 20 days, and then euthanized. The growth and size of the tumors in the mice were observed, and then the tumors were dissected. The tumor weight was measured using a digital balance, and the tumor volume was measured using a ruler. This study was approved by the Animal Ethics Committee of Nanjing Medical University.

### WB assay

Total proteins that were isolated from GC cells and tumor tissues were utilizing RIPA buffer and quantified by the BCA method (P0009, Beyotime, China). 25 µg of proteins were loaded into 10% SDS-PAGE (Servicebio, Wuhan, China) and transferred onto PVDF membranes (Merck Millipore, USA). The membranes were blocked with 5% skim milk and incubated with primary antibodies at 4 °C overnight. Primary antibodies including anti-MT2A (DF6755, 1:1000, Affinity), anti-NOTCH1 (AF5037, 1:1000, Affinity), anti-SLC7A5 (DF8065, 1:1000, Affinity), anti-MMP2 (ab92536, 1:1500, Abcam), anti-MMP9 (ab76003, 1:1000), anti-HK2 (ab209847, 1:1000, Abcam), anti-LDHA (DF6280, 1:1000, Affinity), anti-Glut1 (AF5462, 1:1000, Affinity), and anti-PDK1 (ab207450, 1:1000, Abcam), anti-BCL2 (AF6139, 1:1500, Affinity), anti-Bax (AF0120, 1:1500, Affinity), anti-GAPDH (ab9485, 1:1500, Abcam). Then, the membranes were incubated with goat anti-rabbit IgG (1:10000, Abcam) for 1 h at room temperature and visualized utilizing an ECL kit (Pierce Biotech, Rockford, IL). The bands were analyzed by Image J (Version 1.49, USA).

### Statistical analysis

The data underwent analysis utilizing IBM SPSS 26.0 software (IBM SPSS, Watson, NY, USA) and were subsequently reported in the format of SD. One-way ANOVA accompanied by Tukey’s post hoc test was performed to evaluate distinctions among the experimental groups. Statistical significance was established when the *P* < 0.05.

## Results

### Function enrichment analysis

**Fig. 1:**
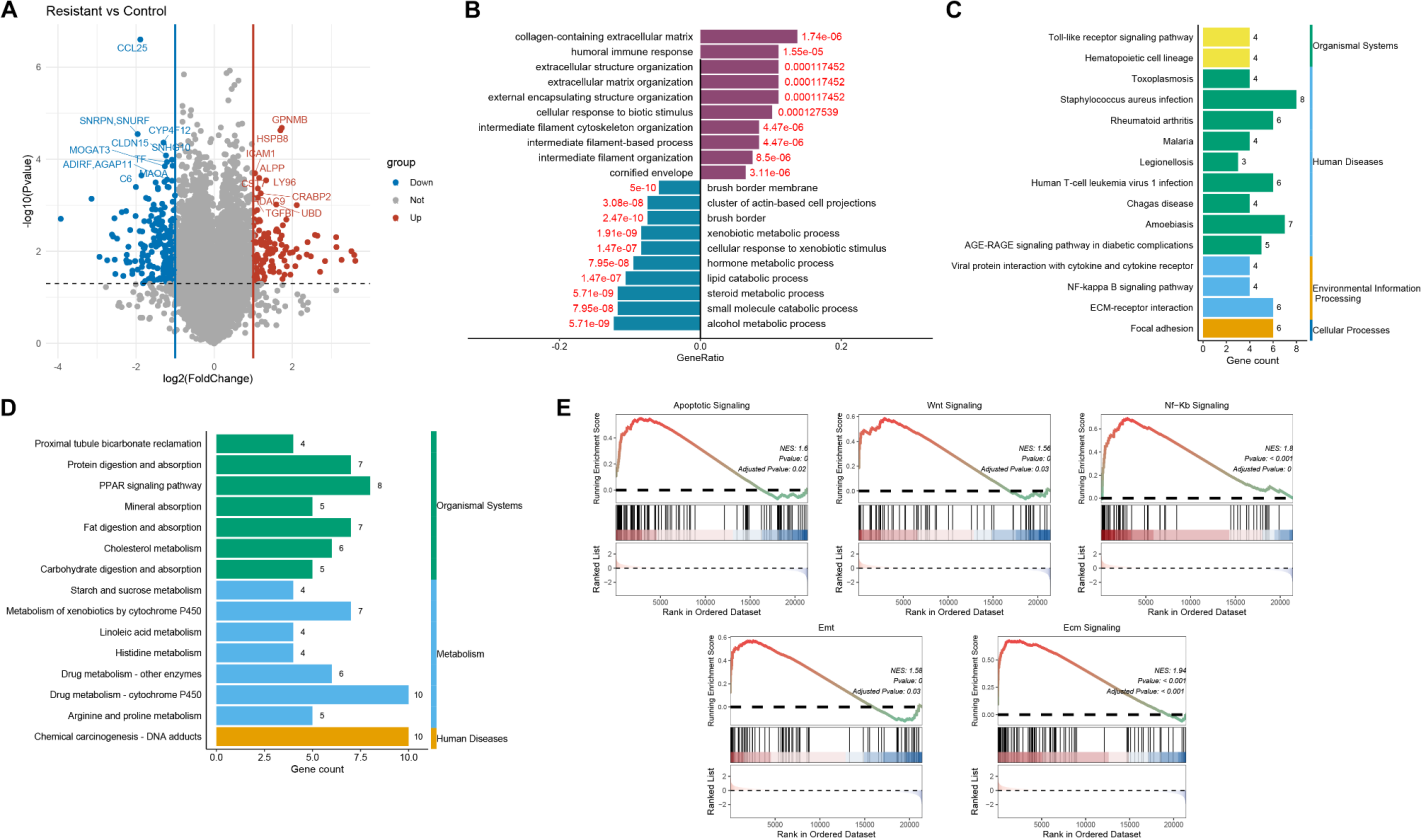
Function enrichment analysis. Note: Volcanic diagram showed differentially expressed genes (DEGs) in GSE128967 dataset (**A**). GO analysis of DEGs (**B**). KEGG analysis of upregulation DEGs (**C**) and downregulation DEGs (**D**). GSEA analysis of platinum resistance-related genes (**E**)

In an effort to identify the oxaliplatin resistance genes, differential analysis identified a total of 316 DEGs (|log2 FC| > 1, *P* < 0.05) in GSE128967 dataset, of which 116 were upregulated and 200 were downregulated (Fig. 1A, S1). GO analysis illustrated that DEGs were enriched in terms such as humoral immune response, collagen containing extracellular matrix and xenobiotic metabolic process (Fig. 1B). KEGG results illustrated that upregulation DEGs were mainly enriched in toll-like receptor signaling pathway, NF-kappa B signaling pathway, ECM-receptor interaction (Fig. 1C, *P* < 0.05), downregulation DEGs were mainly enriched in PPAR signaling and Metabolism of xenobiotics by cytochrome P450 (Fig. 1D, *P* < 0.05). GSEA analysis showed that PRGs were mainly enriched in apoptotic, Ecm, Wnt, NF-kappa B, and EMT signaling (Fig. 1E, adj. *P* < 0.05).

### Identification of oxaliplatin-resistance related genes with prognostic value in GC

**Fig. 2:**
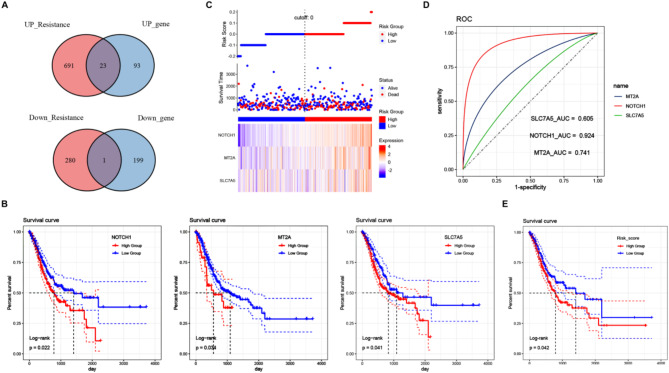
Identification of oxaliplatin resistance related genes with prognostic value in GC. Note: Venn diagram showing the intersection of GSE128967 dataset and platinum resistance-related genes (**A**). Kaplan-Meier (KM) survival curves for MT2A, NOTCH1 and SLC7A5 (**B**). Triple plot of risk score (**C**). The ROC curve showed the contribution of MT2A, NOTCH1 and SLC7A5 to the risk score (**D**). KM survival curve of patients in high-risk (HR) group and low-risk (LR) group after grouping according to risk score (**E**)

Twenty-four OXARGs in GC were initially obtained by intersecting DEGs with PRGs (Fig. 2A, S2). The KM curves of these 24 genes were plotted based on survival data in TCGA-STAD, and the results showed that MT2A, NOTCH1 and SLC7A5 had a significant effect on patient survival (Fig. 2B). Risk score was determined based on the expression of the 3 genes, and patients in TCGA-STAD were classified into high-score (HS) and low-score (LS) groups (Fig. 2C). Figure 2D showed the degree of contribution of MT2A, NOTCH1 and SLC7A5 to the risk score, with NOTCH1 contributing the most to the risk score. Patients in the HS group had a shorter overall survival time than in the LS group (Fig. 2E), indicating that the risk score significantly influenced survival.

### Immune infiltration analysis

**Fig. 3:**
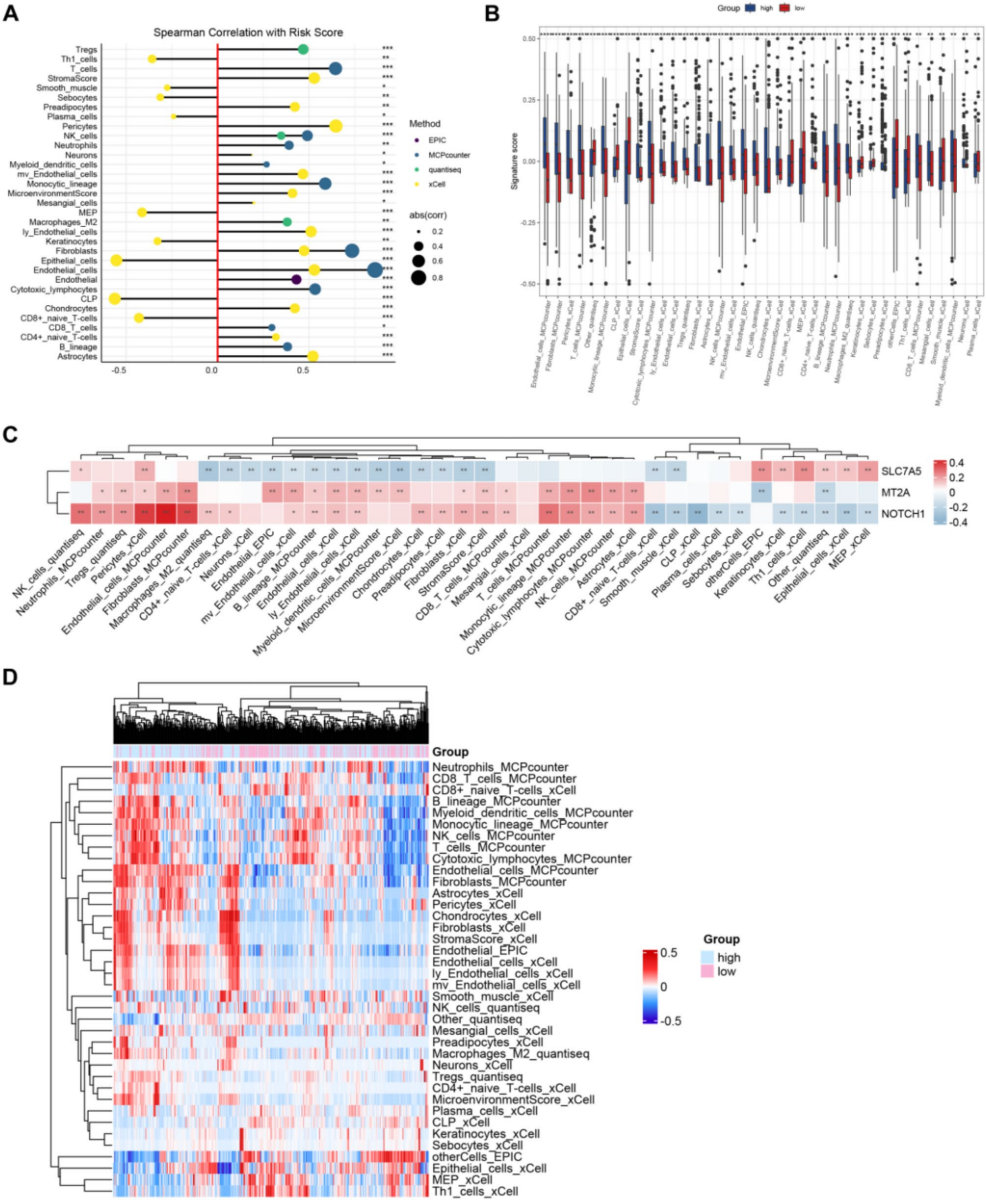
Immune infiltration analysis. Note: Correlation between immune infiltration score and risk score (**A**). Comparison of immune cell infiltration between high score (HS) group and low score (LS) group (**B**). The correlation between the 3 prognostic genes and immune cells (**C**). The immunity score heat map visualized the correlation between immune cells and risk score (**D**)

Next, we analyzed the correlation between risk score and immune cells. The results demonstrated that risk score was significantly correlated with a wide range of immune cells, especially positively correlated with cells favoring immunosuppression, such as Treg and M2 macrophages (Fig. 3A). Also, the immune cell infiltration was significantly different between GC patients in the HS and LS groups, and patients in the HS group had reduced infiltration of anticancer immune cells, such as CD8 + naive T cells, common lymphoid progenitor (CLP) cells, and plasma cells, suggesting that patients in the HS group had reduced immune response capacity (Fig. 3B). Moreover, we analyzed the relationship between the 3 prognostic genes and immune cells (Fig. 3C). The results showed that SLC7A5 had a significant positive correlation with NK cells, keratinocytes and MEP cells, and a significant negative correlation with macrophages_M2, CD4+_naive_T cells. MT2A had a significant positive correlation with fibroblasts, cytotoxic_lymphocytes, NK_cells. NOTCH1 was positive correlation with NK_cells, pericytes, preadipocytes, T_cells, and negative correlation with CD8+_naive_T-cells, plasma_cells, Th1_cells. The immunity score heat map exhibited that there was a significant difference in the immune immunity scores of different immune cells between the HS and LS groups (Fig. 3D).

### CNV mutation and drug sensitivity analyses

**Fig. 4:**
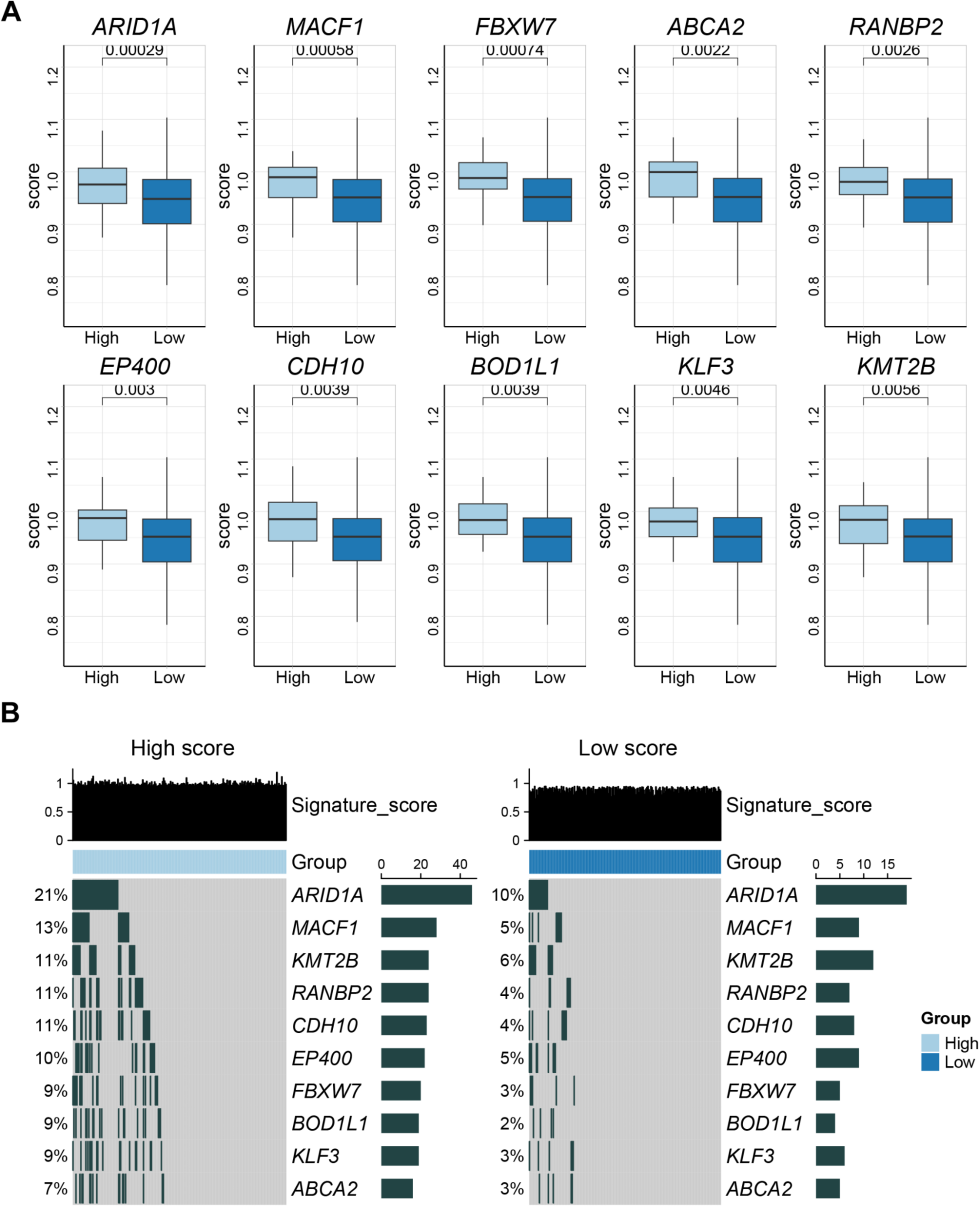
CNV mutation analysis. Note: The top 10 genes with the most significant mutations in high score (HS) group and low score (LS) group (**A**). Oncoplots showed the level of CNV mutations in HS and LS groups (**B**)

Mutation analysis revealed a total of 18 significantly mutated genes between the HS and LS groups (S3, *P* < 0.01), and the top 10 genes with the most significant mutations were shown in Fig. 4A. Oncoplots (Fig. 4B) illustrated that the level of CNV mutations in the HS group was significantly higher than in the LS group, with the most significant difference being in ARID1A (21%/10%).

**Fig. 5:**
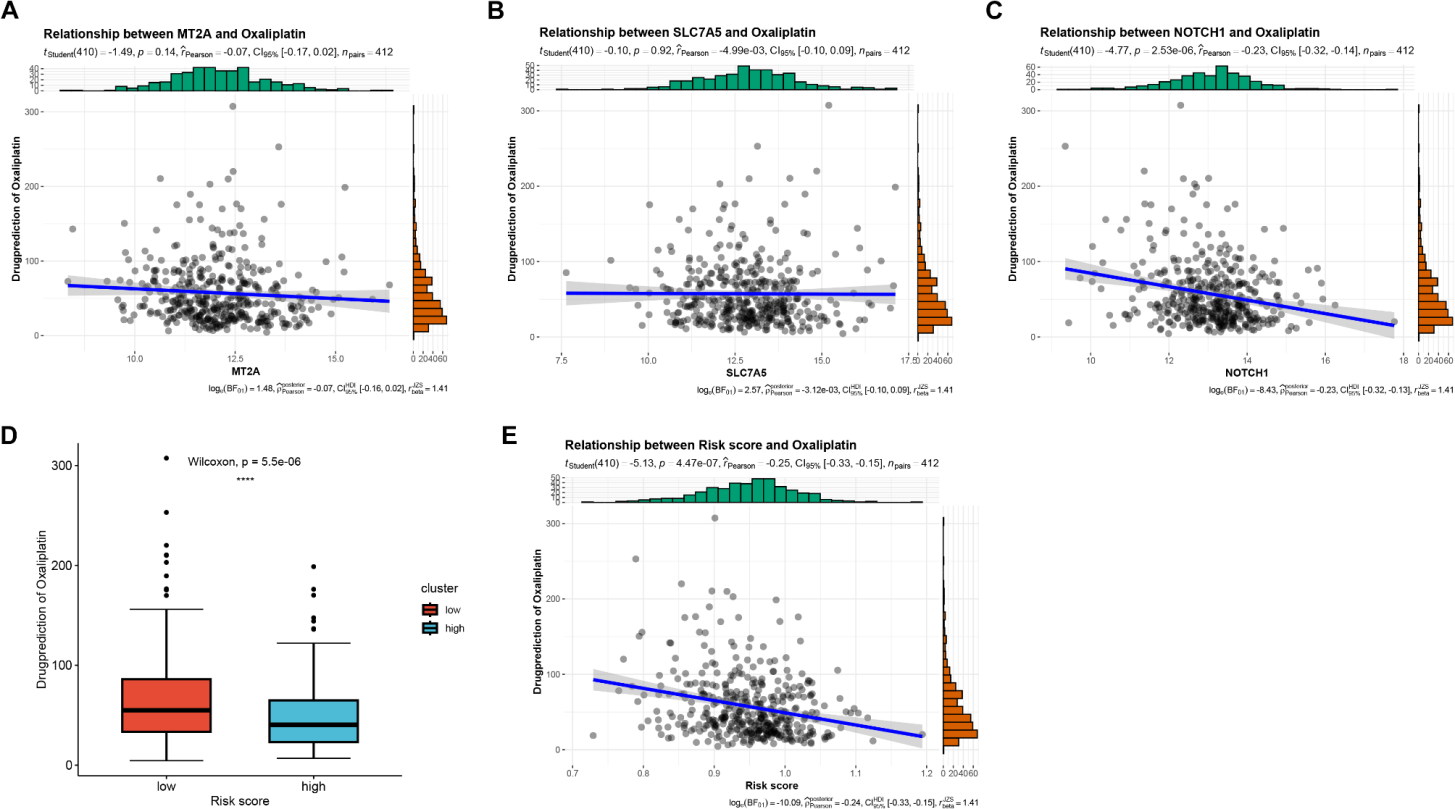
Drug-susceptibility Analysis. Note Correlation of MT2A (**A**), SLC7A5(**B**) and NOTCH1(**C**) with drug sensitivity. High score (HS) group and low score (LS) group of drug sensitivity boxplot (**D**). Correlation between risk score and drug sensitivity (**E**)

The drug sensitivities of MT2A (Fig. 5A), SLC7A5 (Fig. 5B), and NOTCH1 (Fig. 5C) were predicted in this study using the pan-cancer database of oncoPredict. The results demonstrated that only NOTCH1 was significantly negatively correlated with drug sensitivity. MT2A and SLC7A5 may be oxaliplatin-resistant genes unique to GC and thus were not significantly correlated with drug sensitivity in the pan-cancer database. In addition, drug sensitivity was higher in the LS group than in the HS group (Fig. 5D), and risk score was significantly negatively correlated with drug sensitivity (Fig. 5E).

### Validation the expression of MT2A, NOTCH1 and SLC7A5 in oxaliplatin-resistant GC cells

**Fig. 6:**
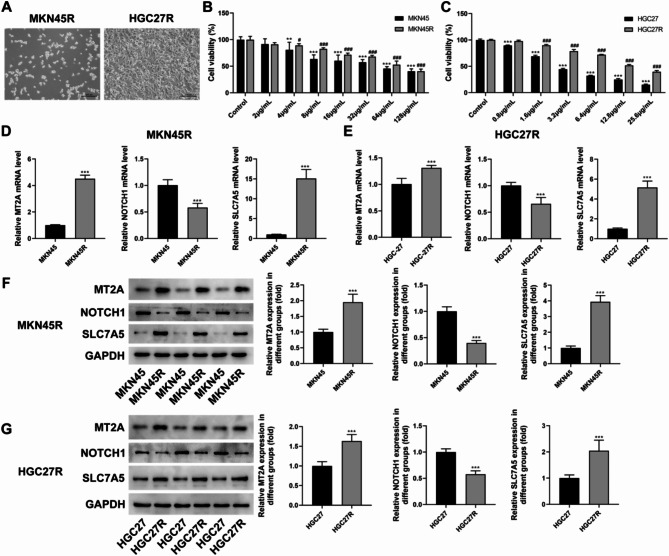
Validation the expression of MT2A, NOTCH1 and SLC7A5 in oxaliplatin (oxaliplatin)-resistant gastric cancer (GC) cells. Note: MKN45R and HGC27R cells (**A**). Effect of different doses of oxaliplatin on the viability of MKN45 and MKN45R cells, *P < 0.05, ***P < 0.001 vs. Control (**B**). Effect of different doses of oxaliplatin on the viability of HGC27 and HGC27R cells, ***P < 0.001 vs. Control (**C**). The mRNA levels of MT2A, NOTCH1, and SLC7A5 in MKN45, MKN45R, HGC27 and HGC27R, ***P < 0.001 vs. MKN45 or HGC27 (**D**-**E**). Expression of MT2A, NOTCH1 and SLC7A5 proteins in MKN45 and MKN45R cells, ***P < 0.001 vs. MKN45 (**F**). Expression of MT2A, NOTCH1 and SLC7A5 proteins in HGC27 and HGC27R cells, ***P < 0.001 vs. HGC27 (**G**). Each experiment was repeated three times

The results of bioinformatics analysis illustrated that the risk score better reflected the prognosis, immune infiltration, CNV mutation and drug sensitivity of GC patients, indicating that the MT2A, NOTCH1 and SLC7A5 were the potential targets for oxaliplatin resistance in GC. Next, we validated the expression of MT2A, NOTCH1 and SLC7A5 in two oxaliplatin-resistant GC cells, MKN45R and HGC27R (Fig. 6A). CCK8 results showed that MKN45R cells were resistant to 24 h of oxaliplatin treatment at a concentration of about 8 µg/mL with a resistance index of 1.6 (Fig. 6B). The HGC27R cells were resistant at a dose of 1. 6 µg/mL with a resistance index of 4.5 (Fig. 6C). RT-qPCR (Fig. 6D, E) and WB (Fig. 6F, G) results indicated an increase in the expression levels of MT2A and SLC7A5 and a significant reduction in the expression level of NOTCH1 in the oxaliplatin-resistant cells compared to MKN45 and HGC27 cells. However, studies have shown that MT2A is an oncogene (Pan et al. 2013, 2016), which is inconsistent with the results of our analysis. In addition, the role of NOTCH1 in GC has been more extensively reported. Therefore, SLC7A5 was initially included in this follow-up study.

### Knockdown of SLC7A5 inhibited the malignant processes of MKN45R and HGC27R cells

**Fig. 7:**
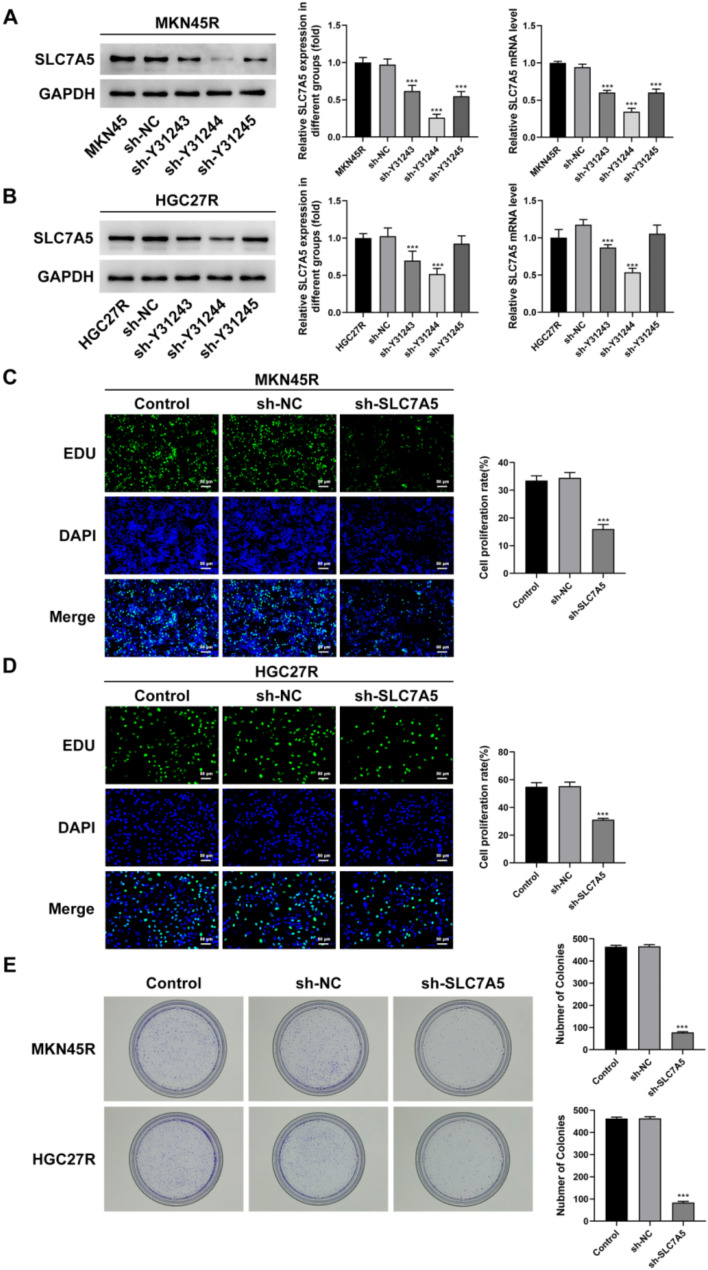
Knockdown of SLC7A5 inhibited the proliferation of MKN45R and HGC27R cells. Note: Detection of interference efficiency of three sh-SLC7A5 in MKN45R cells, ***P < 0.001 vs. MKN45R (**A**). Detection of interference efficiency of three sh-SLC7A5 in HGC27R cells, ***P < 0.001 vs. HGC27R (**B**). EDU staining detected the effect of sh-SLC7A5 on proliferation of MKN45R cells, ***P < 0.001 vs. sh-NC (**C**). EDU staining detected the effect of sh-SLC7A5 on proliferation of HGC27R cells, ***P < 0.001 vs. sh-NC (**D**). Colony formation assay detected the effect of sh-SLC7A5 on the ability of HGC27R and MKN45R cells colony formation, ***P < 0.001 vs. sh-NC (**E**).Each experiment was repeated three times

**Fig. 8:**
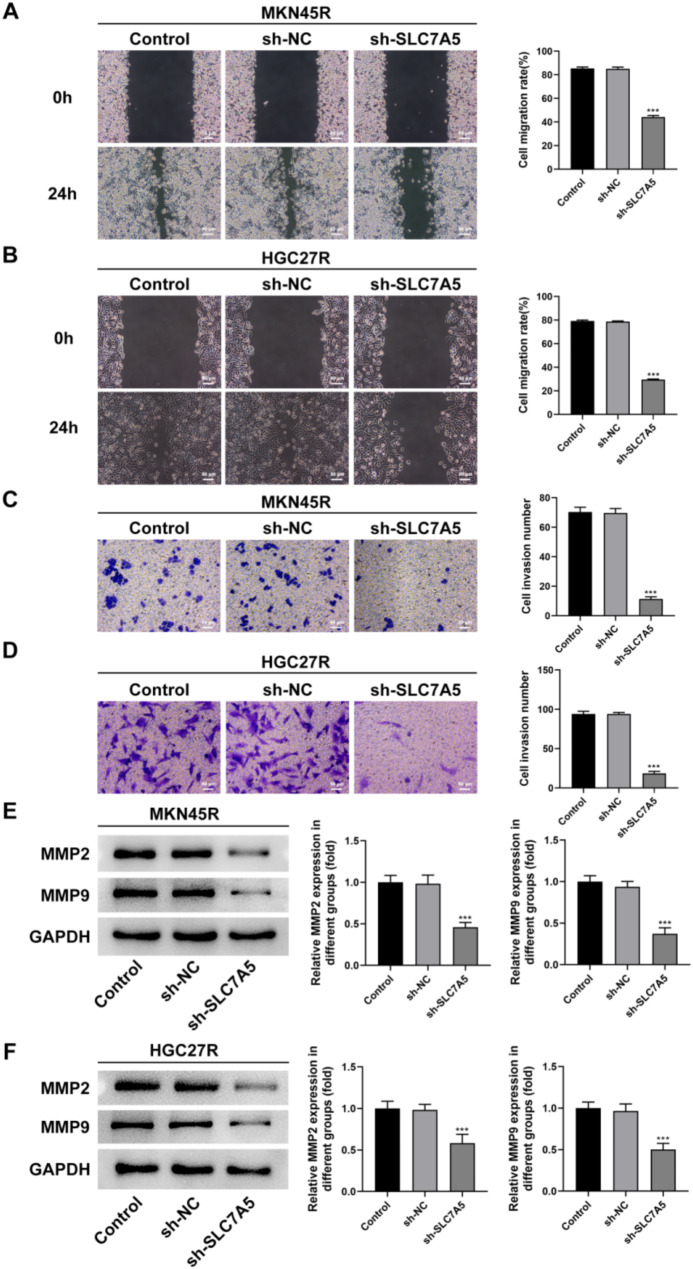
Knockdown of SLC7A5 inhibited the invasion and migration of MKN45R and HGC27R cells. Note: Wound healing assay detected the effect of sh-SLC7A5 on the migration of MKN45R cells (**A**) and HGC27R cells (**B**). Transwell assay detected the effect of sh-SLC7A5 on the invasion of MKN45R cells (**C**) and HGC27R cells (**D**). Western blot assay detected the effect of sh-SLC7A5 on the expression of MMP2 and MMP9 in MKN45R cells (**E**) and HGC27R cells (**F**). ***P < 0.001 vs. sh-NC. Each experiment was repeated three times

**Fig. 9:**
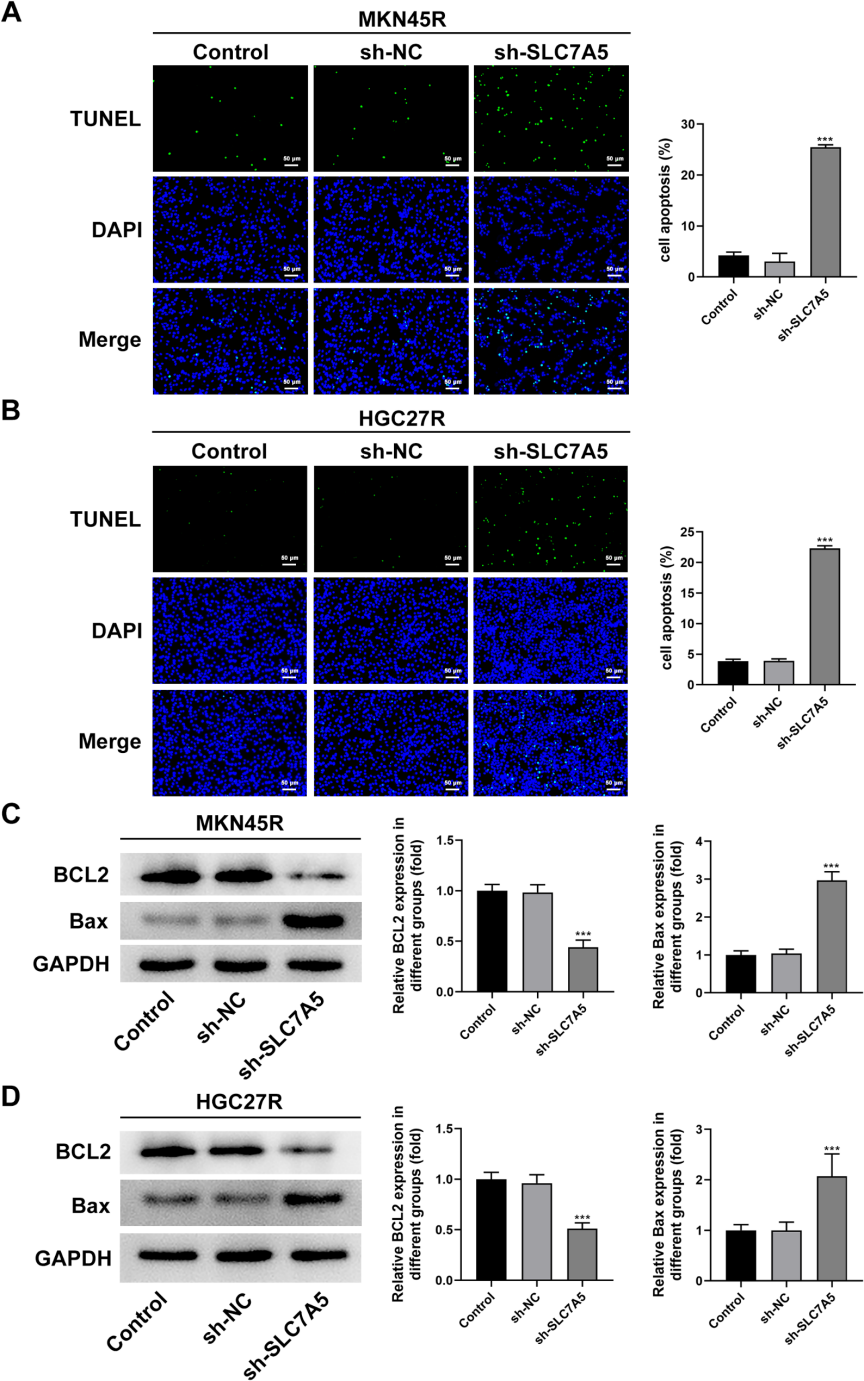
Knockdown of SLC7A5 promoted the apoptosis of MKN45R and HGC27R cells. Note: TUNEL staining detected the effect of sh-SLC7A5 on the apoptosis of MKN45Rcells (**A**) and HGC27R cells (**B**). Western blot assay detected the effect of sh-SLC7A5 on the expression of BCL2 and Bax in MKN45R cells (**C**) and HGC27R cells (**D**). ***P < 0.001 vs. sh-NC. Each experiment was repeated three times

The sh-SLC7A5 was used to explore the role of SLC7A5 in oxaliplatin-resistant GC cells. The results showed that all three sh-SLC7A5 reduced the expression of SLC7A5 in MKN45R (Fig. 7A) and HGC27R (Fig. 7B) cells, among which the Y31244 target had the best interference effect and was selected for further experiments. The results of EDU staining and cloning experiments showed that the fluorescence intensity of GC cells in the sh-SLC7A5 group was significantly reduced compared with sh-NC group (Fig. 7C, D), and the cell cloning ability was significantly decreased (Fig. 7E). In addition, cell migration ability (Fig. 8A, B) and invasion ability (Fig. 8C, D) were significantly attenuated in the sh-SLC7A5 group, and the expression of MMP2 and MMP9 was significantly downregulated (Fig. 8E, F). The result of TUNEL staining exhibited that the fluorescence intensity of MKN45R (Fig. 9A) and HGC27R (Fig. 9B) cells in the sh-SLC7A5 group was enhanced WB resulted demonstrated that Bcl-2 expression was significantly downregulated and Bax expression was obviously increased in the sh-SLC7A5 group (Fig. 9C, D).

### Knockdown of SLC7A5 inhibited the glycolysis of MKN45R and HGC27R cells

**Fig. 10:**
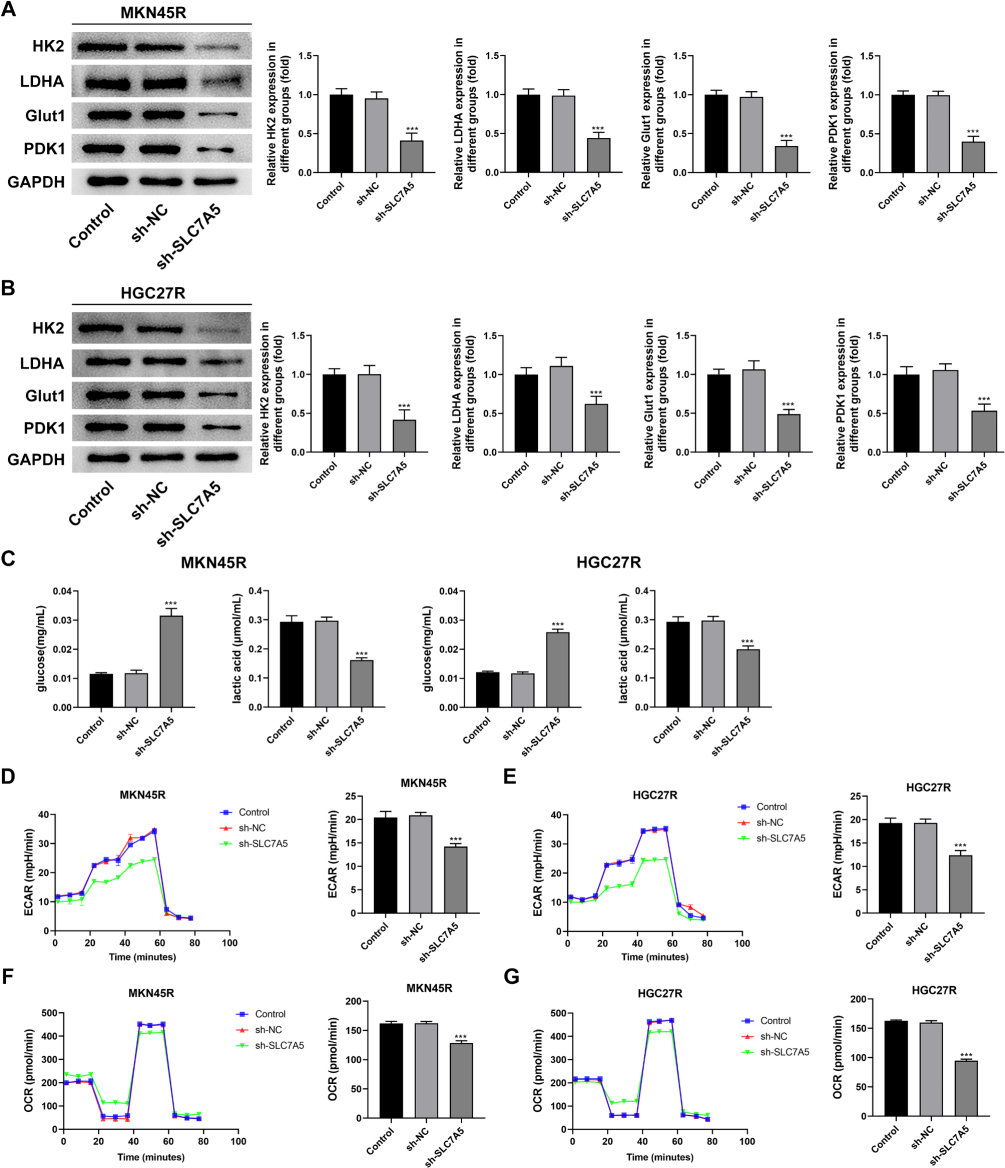
Knockdown of SLC7A5 inhibited the glycolysis of MKN45R and HGC27R cells. Note: Western blot assay detected the effect of sh-SLC7A5 on the expression of HK2, LDHA, Glut1, PDK1 in MKN45R cells (**A**) and HGC27R cells (**B**). Enzyme-linked immunosorbent assay detection extracellular glucose levels and extracellular lactic acid level (**C**). Effects of sh-SLC7A5 on extracellular acidification rate (ECAR) in MKN45R cells (**D**) and HGC27R cells (**E**). Effects of sh-SLC7A5 on oxygen consumption rate (OCR) in MKN45R cells (**F**) and HGC27R cells (**G**). ***P < 0.001 vs. sh-NC. Each experiment was repeated three times

The Warburg effect observed in tumor cells is characterized by increased glucose uptake and heightened glycolytic activity. This phenomenon is intricately linked to the development, spread, metastasis, resistance to treatment, and unfavorable prognosis of gastric cancer (Liu et al. 2019). Thus, we examined the effect of SLC7A5 knockdown on the expression of glucose transport-related proteins in HGC27R cells and MKN45R cells. The results indicated that knockdown of SLC7A5 significantly reduced the expression of HK2, LDHA, Glut1, and PDK1 in MKN45R cells (Fig. 10A) and HGC27R cells (Fig. 10B). In addition, knockdown of SLC7A5 resulted in a significant increase in glucose levels and a significant reduce in lactate levels in the supernatants of MKN45R and HGC27R cells (Fig. 10C). Finally, knockdown of SLC7A5 significantly decreased the ECAR (Fig. 10D, E) and OCR (Fig. 10F, G) in MKN45R and HGC27R cells. The results indicated that knockdown of SLC7A5 significantly reduced mitochondrial respiratory reserve capacity and inhibited glycolysis in MKN45R and HGC27R cells.

### Knockdown of SLC7A5 inhibited the malignant processes of MKN45R and HGC27R cells by suppressing Glycolysis

**Fig. 11:**
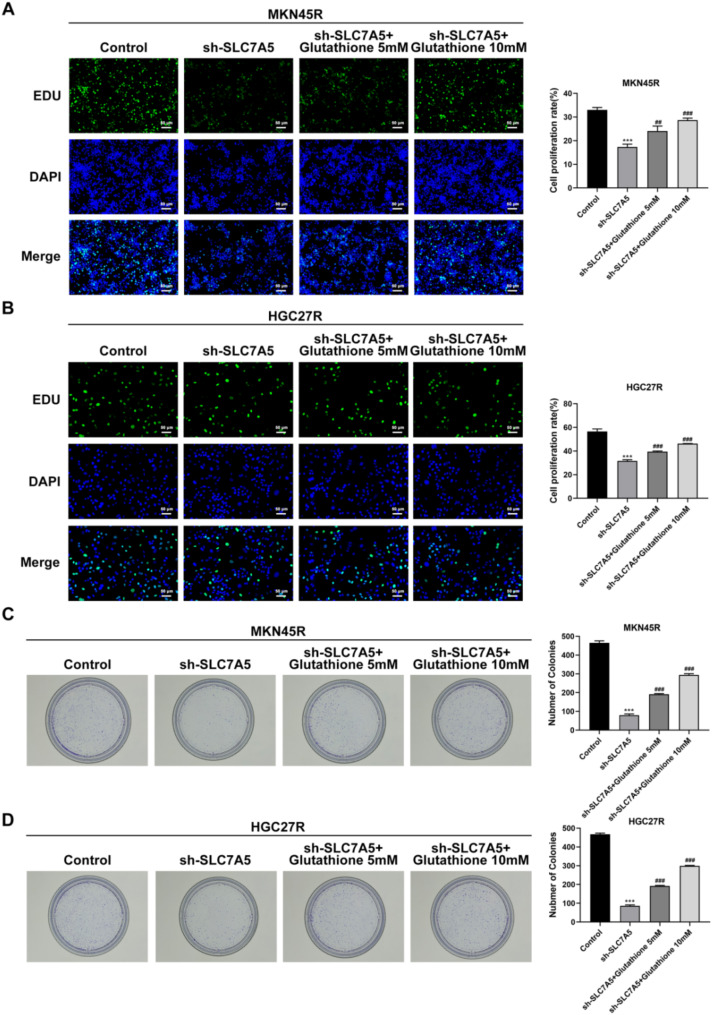
Knockdown of SLC7A5 inhibited the proliferation of MKN45R and HGC27R cells by suppressing glycolysis. Note: EDU staining detected the proliferation of MKN45R cells (**A**) and HGC27R cells (**B**). Colony formation assay detected the colony formation ability of MKN45R cells (**C**) and HGC27R cells (**D**). ***P < 0.001 vs. Control; ^##^*P* < 0.01, ^###^*P* < 0.001 vs. sh-SLC7A5. Each experiment was repeated three times

**Fig. 12:**
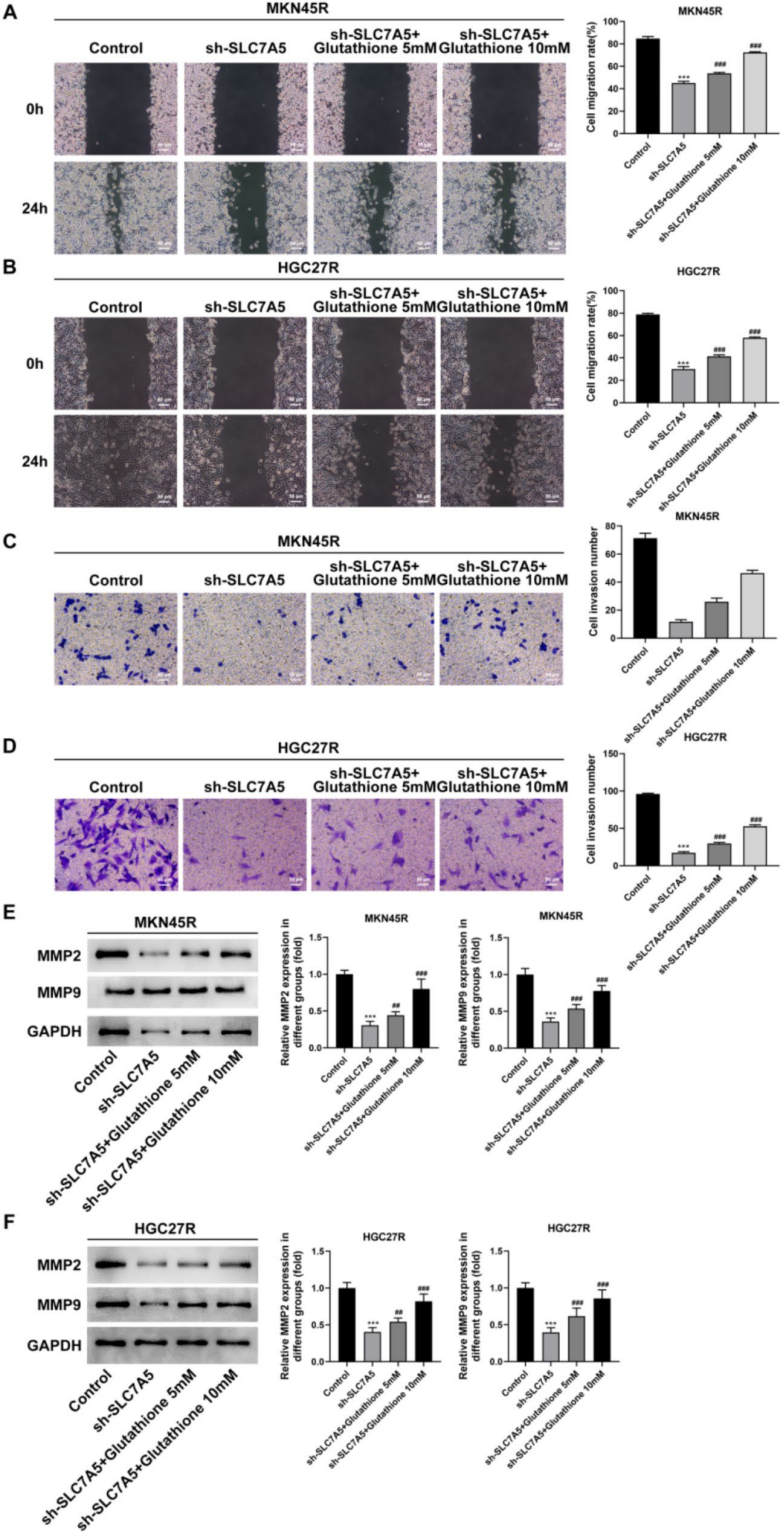
Knockdown of SLC7A5 inhibited the invasion and migration of MKN45R and HGC27R cells by suppressing glycolysis. Note: Wound healing assay detected the migration of MKN45R cells (**A**) and HGC27R cells (**B**). Transwell assay detected the effect invasion of MKN45R cells (**C**) and HGC27R cells (**D**). Western blot assay detected the expression of MMP2 and MMP9 in MKN45R cells (**E**) and HGC27R cells (**F**). ***P < 0.001 vs. Control; ^##^*P* < 0.01, ^###^*P* < 0.001 vs. sh-SLC7A5. Each experiment was repeated three times

**Fig. 13:**
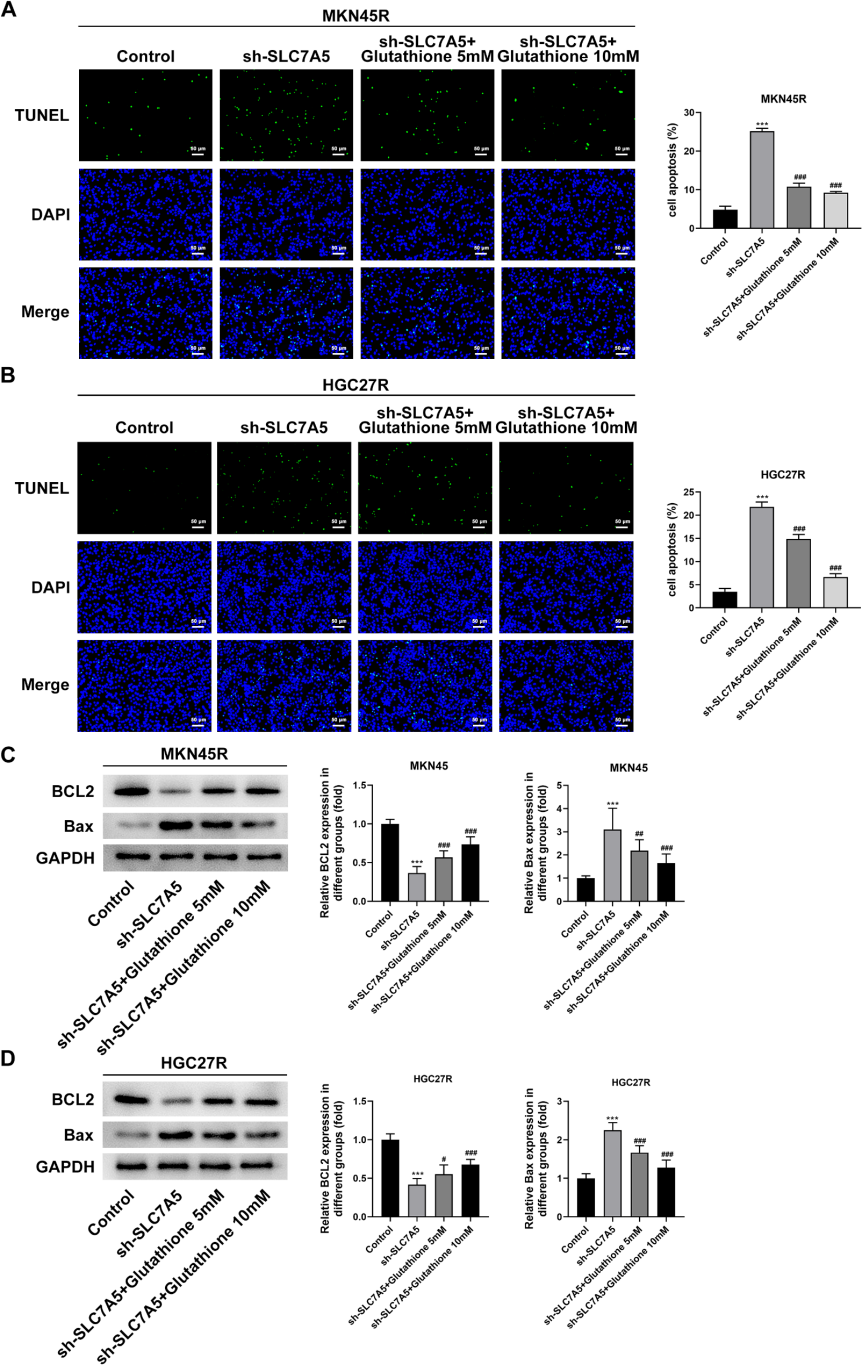
Knockdown of SLC7A5 promoted the apoptosis of MKN45R and HGC27R cells by suppressing glycolysis. TUNEL staining detected the apoptosis of MKN45R cells (**A**) and HGC27R cells (**B**). Western blot assay detected the expression of BCL2 and Bax in MKN45R cells (**C**) and HGC27R cells (**D**). ***P < 0.001 vs. Control; ^#^*P* < 0.05, ^##^*P* < 0.01, ^###^*P* < 0.001 vs. sh-SLC7A5. Each experiment was repeated three times

To clarify whether the effect of sh-SLC7A5 on the malignant progression of oxaliplatin-resistant GC cells was related to the inhibition of glycolysis, different doses of glutathione were used in the follow-up study. The results illustrated that the addition of 5 mM and 10 mM glutathione significantly enhanced the proliferation (Fig. 11A, B), clone formation (Fig. 11C, D), migration (Fig. 12A, B), and invasion (Fig. 12C, D) abilities of MKN45R and HGC27R cells compared with the sh-SLC7A5 group, and upregulated the expression of MMP2 and MMP9 (Fig. 12E, F). Also, glutathione significantly decreased the apoptosis level of MKN45R cells (Fig. 13A) and HGC27R cells (Fig. 13B), upregulated the expression of Bcl-2 and downregulated the expression of Bax (Fig. 13C, D).

### Knockdown of SLC7A5 inhibited the growth and glycolysis-related proteins expression of MKN45R and HGC27R cells in vivo

**Fig. 14:**
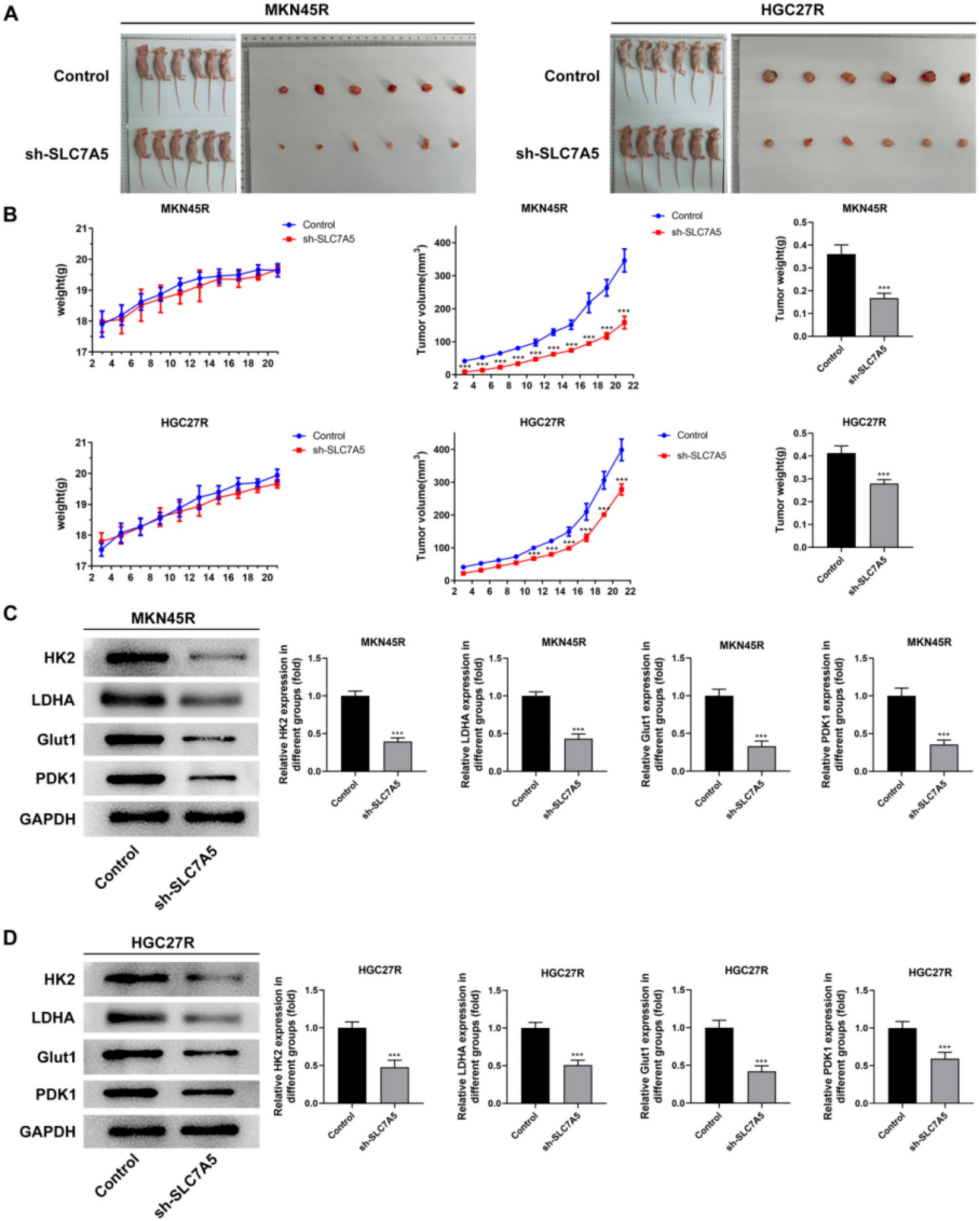
Knockdown of SLC7A5 inhibited the growth and glycolysis-related proteins expression of HGC27R and MKN45R cells in vivo. Note: Tumor tissue size of mice in different treatment groups (**A**). Body weight, tumor tissue volume, and tumor weight of mice in different treatment groups (**B**). The expression of glucose transport-related proteins HK2, LDHA, Glut1, PDK1 in tumor tissues of mice in MKN45R cells (**C**) and HGC27R cells (**D**). ***P < 0.001 vs. Control. Each experiment was repeated three times

The results of the vivo experiment showed that the tumor volume and weight of mice in the sh-SLC7A5 group were significantly reduced (Fig. 14A, B), indicating that knocking down SLC7A5 inhibited the growth of MKN45R and HGC27R cells in vivo. In addition, the expression levels of HK2, LDHA, Glut1, and PDK1 were significantly decreased in the tumor tissues of MKN45R (Fig. 14C) and HGC27R (Fig. 14D) cells in the sh-SLC7A5 group.

### Knockdown of SLC7A5 enhanced the sensitivity of MKN45R and HGC27R cells to oxaliplatin in vitro and in vivo

**Fig. 15:**
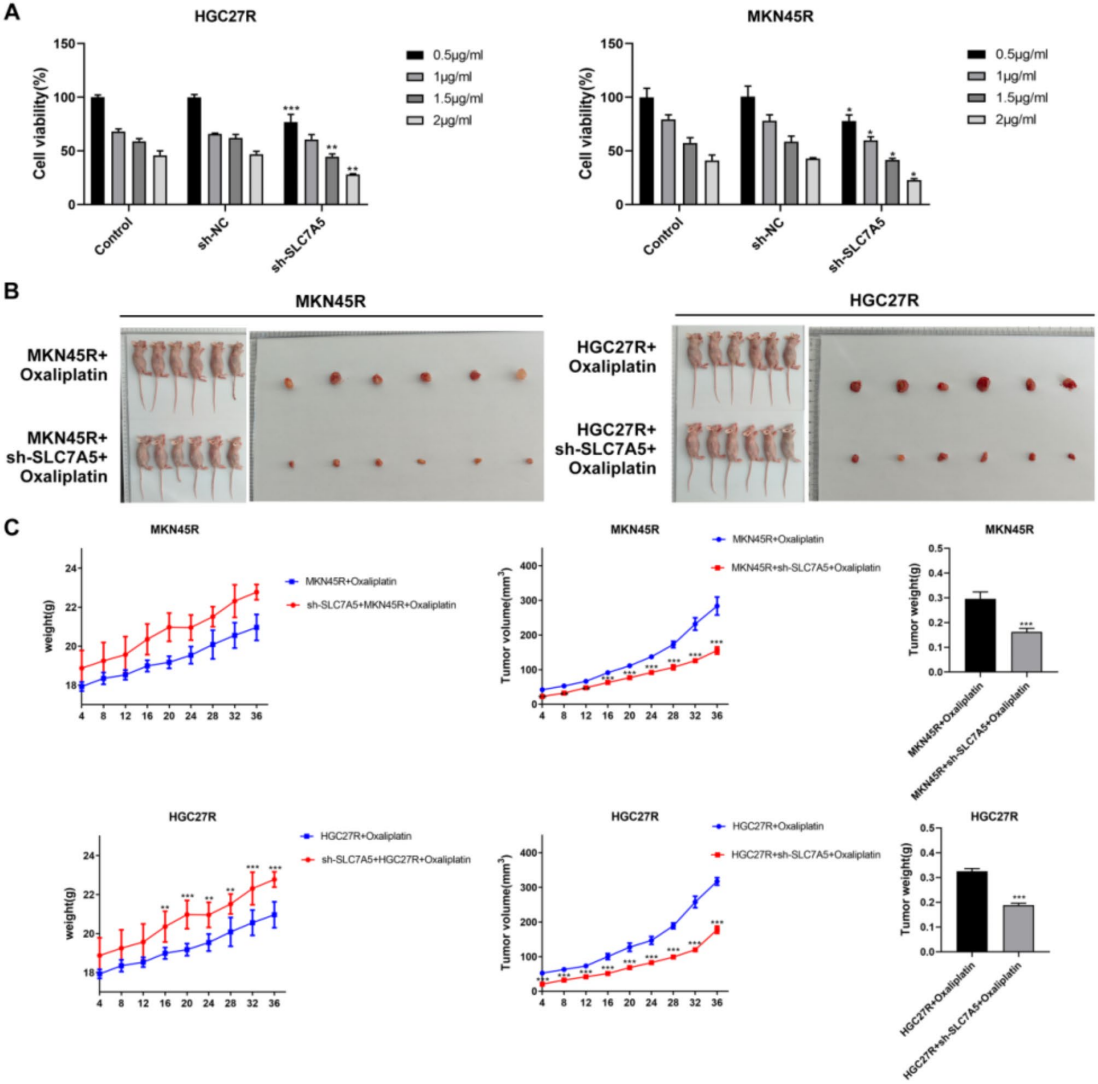
Knockdown of SLC7A5 enhanced the sensitivity of HGC27R and MKN45R cells to oxaliplatin in vitro and in vivo. Note: Effect of different doses of oxaliplatin on the viability of HGC27R and MKN45R cells, *P < 0.05, **P < 0.01, ***P < 0.001 vs. sh-NC (**A**). Tumor tissue size of mice in different treatment groups (**B**). Body weight, tumor tissue volume, and tumor weight of mice in different treatment groups, *P < 0.05, **P < 0.01, ***P < 0.001 vs. HGC27R + Oxaliplatin or MKN45R + Oxaliplatin (**C**). Each experiment was repeated three times

Finally, we observed the sensitivity of HGC27R and MKN45R cells to oxaliplatin after SLC7A5 knockdown. CCK8 results showed that oxaliplatin at 0.5 µg/mL significantly reduced the viability of HGC27R and MKN45R cells after knockdown of SLC7A5 compared with the sh-NC group (Fig. 15A). The resistance indices of HGC27R and MKN45R cells lines after knockdown of SLC7A5 are 0.7 and 0.6 respectively. In vivo, knockdown of SLC7A5 and administration of oxaliplatin significantly reduced tumor volume and weight in mice compared to oxaliplatin alone (Fig. 15C, D). This suggested that the knockdown of SLC7A5 enhanced the sensitivity of MKN45R and HGC27R cells to oxaliplatin in vitro and in vivo.

## Discussion

The emergence of drug resistance has brought significant challenges to the treatment of GC (Deng et al. 2021). This study identified the potential role of SLC7A5 in oxaliplatin resistance in GC through bioinformatics analysis and experimental validation. Further analysis indicated that sh-SLC7A5 significantly suppressed the growth of HGC27R and MKN45R cells in vitro and in vivo by suppressing glycolysis, and enhanced the sensitivity of HGC27R and MKN45R to oxaliplatin.

Utilizing TCGA and GEO datasets to predict and analyze tumor biomarkers has established a crucial theoretical foundation for advancing molecular targeted therapies in cancer (Lu et al. 2021; Ucaryilmaz Metin and Ozcan 2022). This study preliminarily identified the prognostic value and expression of SLC7A5 in GC through bioinformatics analysis and experimental validation. SLC7A5, also known as LAT1, facilitates the transmembrane transport of large neutral amino acids (Bhutia et al. 2015). Cancer cells sustain survival and engage in malignant behaviors such as invasion and migration through substantial amino acid absorption (Markowicz-Piasecka et al. 2020). Studies indicate that SLC7A5 shows significantly elevated expression in various malignant tumors, including GC, colon cancer, lung cancer, and prostate cancer (Wang et al. 2016; Najumudeen et al. 2021), especially has been shown to be a promoter of GC (Wang et al. 2013). Furthermore, studies demonstrated that MicroRNA-126 inhibits GC cell proliferation via targeting SLC7A5 (Wang et al. 2015). CRKL promotes the migration of SGC-7901 cells by regulating SLC7A5 (Wang et al. 2016). Additionally, SLC7A5 activates the mTOR pathway in GC cells, facilitating cell proliferation (Wu et al. 2024). These studies highlight the strong association between elevated SLC7A5 expression and the malignant progression of GC. Our research found that knocking down SLC7A5 markedly suppressed proliferation, invasion, migration, and in vivo growth of oxaliplatin-resistant GC cells, inducing apoptosis. This suggests that SLC7A5 is involved not only in the malignant progression of GC but also significantly correlates with GC resistance to oxaliplatin. To validate this hypothesis further, we knocked down SLC7A5 in oxaliplatin-resistant GC cells and treated them with varying concentrations of oxaliplatin. Results indicated that HGC27R and MKN45R cells exhibited resistance doses of 1.6 µg/mL and 8 µg/mL, respectively, before SLC7A5 knockdown. After knocking down SLC7A5, a concentration of 0.5 µg/mL oxaliplatin significantly decreased the viability of HGC27R and MKN45R cells. Furthermore, in vivo experiments showed that combined sh-SLC7A5 and oxaliplatin treatment more effectively suppressed tumor growth compared to treatment with oxaliplatin alone. These findings emphasize the role of elevated SLC7A5 expression in the development of oxaliplatin resistance in GC.

Glucose is the main energy source for tumor cell growth. Cancer cells promote metastasis and resistance to chemotherapy by increasing glucose uptake and glycolysis, leading to a more acidic tumor environment (Wang et al. 2020a, b; Vander Heiden and DeBerardinis 2017). The upregulation of the glycolytic pathway is not only a metabolic response of cancer cells to adapt to chemotherapy but also one of the key mechanisms of their drug resistance. The Warburg effect, which involves aerobic glycolysis, is closely linked to the aggressive behavior of tumor cells (Yuan et al. 2016; Abbassi-Ghadi et al. 2013). Literature suggests that SLC7A5 enhances glycolytic activity (Kedia-Mehta et al. 2023; Yoon et al. 2018). Moreover, higher glycolysis and ATP production have been observed in colorectal cancer cells resistant to oxaliplatin (Wang et al. 2020a, b). Therefore, does elevated SLC7A5 expression in oxaliplatin-resistant GC cells impact glycolysis, and does SLC7A5 knockdown modulate malignant progression in GC cells through glycolysis. To investigate this question, we assessed glycolysis-related markers in HGC27R and MKN45R cells. HK2, LDHA, Glut1, and PDK1 are crucial proteins involved in glucose transport and glycolysis (Li et al. 2018; Lyu et al. 2018). Our study demonstrated that reducing SLC7A5 expression significantly decreased HK2, LDHA, Glut1, and PDK1 levels in HGC27R cells, MKN45R cells, and tumor tissues. Additionally, SLC7A5 knockdown markedly elevated glucose levels and reduced lactate levels in the supernatant of HGC27R and MKN45R cells, accompanied by decreased OCR and ECAR—key indicators of mitochondrial respiration, glycolysis, and ATP production (Son et al. 2019). These results indicate that suppressing SLC7A5 reduces glycolysis in HGC27R and MKN45R cells, potentially inducing apoptosis in cancer cells due to energy depletion.

Glutathione was used in subsequent experiments to investigate whether downregulating SLC7A5 inhibits the malignant progression of HGC27R and MKN45R cells through glycolysis reduction. Glutathione, an essential intracellular antioxidant, crucially maintains cellular redox balance and mitigates oxidative stress (Diaz-Vivancos et al. 2015). Glutathione facilitates glycolysis by preserving the critical redox environment and protecting glycolytic enzymes from oxidative damage. This support ensures smooth glycolytic processes, thereby enhancing efficient energy production and cellular metabolic activities (Gasmi et al. 2023; Gaffney et al. 2020). In this study, the addition of 5mM and 10mM glutathione significantly mitigated SLC7A5 knockdown’s inhibitory effects on HGC27R and MKN45R cell proliferation, invasion, and migration, suggesting that suppressing SLC7A5 restrains the malignant progression of oxaliplatin-resistant GC cells through glycolysis inhibition.

Furthermore, we found that SLC7A5 is closely related to immune infiltration analysis and CNV mutation, with significant differences in these two functions among gastric cancer patients with different SLC7A5 expressions. We will further discuss the deep mechanistic connections between SLC7A5, immune infiltration analysis, and CNV mutation in our upcoming research. As an important tumor suppressor gene, the mechanism by which ARID1A affects the expression of SLC7A5 will also be a focus of our next research steps.

This study concludes that the SLC7A5 gene was closely related to the development of resistance to oxaliplatin in GC. Suppression of SLC7A5 expression inhibits the proliferation, invasion, migration in vitro and the growth in vivo of oxaliplatin-resistant GC cells. These findings suggest that SLC7A5 could be a worthy biomarker for gastric cancer and a potential target for combating oxaliplatin resistance. Targeting SLC7A5 to inhibit glycolysis enhances the sensitivity to oxaliplatin. This treatment not only suppresses the proliferation of tumor cells but also improves the tumor microenvironment, reducing chemotherapy-related side effects, thereby providing a more effective treatment option for gastric cancer patients. The observed results in HGC27R and MKN45R cells point towards further exploration of the role of SLC7A5 in GC and resistance to oxaliplatin.

## Conclusion

Knockdown of SLC7A5 inhibits malignant progression and attenuates oxaliplatin resistance in GC by suppressing glycolysis.

## Electronic supplementary material

Below is the link to the electronic supplementary material.

Supplementary Material 1

## Data Availability

No datasets were generated or analysed during the current study.
